# NS5A Gene Analysis by Next Generation Sequencing in HCV Nosocomial Transmission Clusters of HCV Genotype 1b Infected Patients

**DOI:** 10.3390/cells8070666

**Published:** 2019-07-02

**Authors:** Maria Concetta Bellocchi, Marianna Aragri, Luca Carioti, Lavinia Fabeni, Rosaria Maria Pipitone, Giuseppina Brancaccio, Maria Chiara Sorbo, Silvia Barbaliscia, Velia Chiara Di Maio, Fabrizio Bronte, Stefania Grimaudo, Walter Mazzucco, Ferdinando Frigeri, Marco Cantone, Antonio Pinto, Carlo Federico Perno, Antonio Craxì, Giovanni Battista Gaeta, Vito Di Marco, Francesca Ceccherini-Silberstein

**Affiliations:** 1 Department of Experimental Medicine, University of Rome Tor Vergata, 00133 Rome, Italy; 2 Health Promotion Sciences, Maternal and Infant Care, Internal Medicine and Medical Specialties (PROMISE) Department, University of Palermo, 90127 Palermo, Italy; 3 Infectious Diseases, Department of Mental and Physical Health and Preventive Medicine, Campania University “Luigi Vanvitelli”, 80138 Naples, Italy; 4 Department of Molecular Medicine, University of Padua, 35121 Padua, Italy; 5 Hematology Division, “San Sebastiano” Hospital, 81100 Caserta, Italy; 6 Hematology Department, National Cancer Institute “Fondazione Pascale”, IRCCS, 80131 Naples, Italy; 7 Department of Microbiology and Clinic Microbiology, University of Milan, 20162 Milan, Italy

**Keywords:** HCV, acute infection, chronic infection, nosocomial transmission, sequencing, NGS

## Abstract

**Background**: The aim of the study was to investigate the intra-host variability through next-generation-sequencing (NGS) of the NS5A-gene in nosocomial transmission-clusters observed in two Italian hospitals among hepatitis C virus (HCV)-genotype-1b infected patients. **Methods**: HCV-sequencing was performed by Sanger-sequencing (NS3 + NS5A + NS5B) and by NGS (NS5A, MiSeq-Illumina) in 15 HCV-1b infected patients [five acute with onco-hematologic-disease and 10 (4/6 acute/chronic) with β-thalassemia]. Resistance-associated-substitutions (RAS) were analysed by Geno2pheno-algorithm. Nucleotide-sequence-variability (NSV, at 1%, 2%, 5%, 10% and 15% NGS-cutoffs) and Shannon entropy were estimated. Phylogenetic analysis was performed by Mega6-software and Bayesian-analysis. **Results**: Phylogenetic analysis showed five transmission-clusters: one involving four HCV-acute onco-hematologic-patients; one involving three HCV-chronic β-thalassemia-patients and three involving both HCV-acute and chronic β-thalassemia-patients. The NS5A-RAS Y93H was found in seven patients, distributed differently among chronic/acute patients involved in the same transmission-clusters, independently from the host-genetic IL-28-polymorphism. The intra-host NSV was higher in chronic-patients versus acute-patients, at all cutoffs analyzed (p < 0.05). Even though Shannon-entropy was higher in chronic-patients, significantly higher values were observed only in chronic β-thalassemia-patients versus acute β-thalassemia-patients (p = 0.01). **Conclusions**: In nosocomial HCV transmission-clusters, the intra-host HCV quasispecies divergence in patients with acute-infection was very low in comparison to that in chronic-infection. The NS5A-RAS Y93H was often transmitted and distributed differently within the same transmission-clusters, independently from the IL-28-polymorphism.

## 1. Introduction

Hepatitis C is a liver disease caused by the hepatitis C virus (HCV). The virus can cause both acute and chronic hepatitis. Globally, an estimated 71 million people have chronic hepatitis C infection [1]. Acute HCV infection is usually defined as having an estimated duration of infection of less than six months [2]. The incubation period for hepatitis C is from two weeks to six months. Since acute HCV infection is usually asymptomatic, very few people are diagnosed during the acute phase. Furthermore, the majority of individuals develop chronic HCV infection having been previously undiagnosed, as the infection may remain asymptomatic for decades until symptoms slowly reveal serious liver damage [3,4]. A significant number of chronically infected individuals will develop cirrhosis or liver cancer [5,6].

HCV is mainly a bloodborne virus, commonly transmitted through injecting drugs, reuse or inadequate sterilization of medical equipment, or the transfusion of unscreened blood and blood products. Cases of HCV transmission in healthcare settings have been reported [7,8,9]. However, reports on transmissions during acute infection diagnosis are still limited [10].

At present, HCV is classified into eight major genotypes and at least 100 subtypes [11,12,13]. The worldwide distribution and prevalence of each HCV genotype (GT) vary depending on geographical region. A recent global survey found that GT 1 (subtypes 1a and 1b predominate in Europe, North America, and Australia) and GT 3 (primarily found in South Asia, particularly the Indian sub-continent) are the most prevalent, accounting for 46% and 30% of all infections, respectively. The GT 2 (prevalent in Western Africa), GT 4 (prevalent in the Middle East and North and Central Africa), GT 5 (prevalent in South Africa) and GT 6 (prevalent in Southeast Asia) accounted for 9%, 8%, 1% and 6%, respectively. The GT 7 (in Central Africa) and GT 8 (from the Punjab, in India) were found in a few individuals [12,14,15]. In Italy, in recent decades, there has been a change in the epidemiology of viral hepatitis thanks to improved hygienic and socio-economic conditions. However, the HCV epidemics in Italy is still the highest [16] among Western European countries, with GT 1 (a and b subtypes) the most prevalent (with a different prevalence 65–45% according to the human immunodeficiency virus [HIV] coinfection status: HIV-/HIV+), followed by GT 3 (about 19–36%, HIV-/HIV+), GT 2 and GT 4 (about 8–3% and 9–11%, HIV-/HIV+ respectively) and finally GT 5 (about <1%) [17,18,19,20]. 

At intra-host level, HCV is genetically diverse and exists as a quasispecies defined by several closely related yet genetically distinct populations [21]. The extremely high intrinsic genetic variability of HCV is a consequence of its high mutational rate and replication turnover, along with a lack of proof-reading activity of viral polymerase. As a result of this phenomenon, a natural emergence of resistance associated variants (RAVs) against direct acting antivirals (DAA) can arise and may compromise treatment [22]. This is a particular characteristic of HCV [23], although less shared by other viruses responsible for chronic infections, such as hepatitis B virus (HBV) or HIV-1, which are more associated with resistance transmission rather than natural resistance [24,25].

Host genetic factors play a crucial role in spontaneous and treatment-induced HCV infection clearance. Human single nucleotide polymorphisms (SNP) of IL28B gene (CC variant) are considered as a very good predictor of viral clearance [26,27,28,29].

Viral genomic population sequencing, combined with a phylogenetic analysis, is a very useful methodology to investigate epidemiology, evolution, resistance, outbreaks, and transmission of viruses [10,30,31]. Recent advances in the nucleic acid sequencing technologies have begun to replace population Sanger sequencing both for research purposes and in the clinical routine, also for drug resistance testing [32]. Next-Generation Sequencing (NGS) allows for accurate characterization of intra-host HCV populations, detecting low frequency variants and rare mutations [33].

Since the whole genome analysis is expensive and highly complex, the sequencing of specific HCV genomic regions, like Core and/or NS5B, represented, and still represents a more convenient method to study HCV genetic diversity [34]. Furthermore, various studies have showed that performing the sequencing of NS3 and NS5A non-structural genes involved in DAA drug resistance also produces highly reliable genotype information and can therefore provide both resistance and evolutionary information for molecular epidemiology [9,10,34,35].

By performing phylogenetic analysis on NS3, NS5A and NS5B genes using Sanger sequencing, we recently identified separate episodes of HCV transmission in two healthcare settings: one in patients with acute HCV diagnosis affected by onco-hematologic disease [36] and the second in patients with acute HCV diagnosis with β-thalassemia [31]. In this present study, through NS5A NGS analysis we aimed to investigate the intra-host viral quasispecies composition and resistance transmission in 15 patients, all putatively involved in transmission clusters.

## 2. Materials and Methods

### 2.1. Study Population

Serum samples from 28 HCV infected patients followed in two Italian hospitals were analyzed using the Sanger genotypic-resistance-test for NS3, NS5A and NS5B genes. All the patients were naïve to DAAs, with the exception of 1 patient with chronic infection who failed a DAA based regimen. Twenty-two patients were affected by β-thalassemia Major and 6 patients had an onco-hematologic disease [31,36]. Eleven β-thalassemia patients received a diagnosis of acute HCV infection during an outbreak between April and November 2016. Five of these received a diagnosis of de-novo acute infection documented by an increase in ALT values greater than 10 upper normal values, positive serum HCV-RNA and seroconversion from anti-HCV negative to anti-HCV positive, while 6 patients received a diagnosis of HCV reinfection since they had past evidence of HCV infection with spontaneous clearance or sustained virological response to Interferon-based therapy. Eleven β-thalassemia patients had a known chronic HCV infection documented by a persistence of serum HCV-RNA positivity. The six patients with hematological malignances received a diagnosis of de-novo acute HCV infection between January and February 2016. None of these patients had developed anti-HCV antibodies (also at the last follow-up visit), confirming their profound immunodepression state as a result of the disease and the chemotherapy received [36]. All subjects gave their informed consent for inclusion before they participated in the study. The study was conducted in accordance with the Declaration of Helsinki, and the protocol was approved by the Ethics Committee of 22 November 2018 (Project identification code: DASOE/6/prot 86605).

### 2.2. HCV Sanger Sequencing 

The serum of 28 patients was tested for HCV NS3, NS5A and NS5B genotypic resistance test by standard Sanger sequencing, following the procedure described previously [37].

Resistance associated substitutions (RAS) were analysed according to Sorbo et al. Drug Resist Update 2018 [38] and Geno2pheno algorithm [39].

### 2.3. HCV Next Generation Sequencing

NS5A NGS on MiSeq platforms (Illumina Inc, San Diego, CA, USA) was performed according to Nextera XT DNA Library Kit (Illumina Inc) in 15 patients, 5 acute patients with onco-hematologic disease and 10 patients (4 acute; 6 chronic) with β-thalassemia.

In brief, to prepare indexed libraries from each sample for sequencing, the HCV-RNA genomic was subjected on a NS5A-RT-PCR with SuperScript III One-Step RT-PCR System (Invitrogen Life Technologies, Carlsbad, CA, USA) and when necessary on a Nested-PCR with AmpliTaq Gold DNA Polymerase (Applied Biosystems, Foster City, CA, USA). For each sample, 1 ng of amplicon was involved in a tagmentation reaction using a Nextera XT DNA Library Kit (Illumina Inc) and a unique combination of an i5 and an i7 index primer was then added to each tagmented DNA sample. The libraries were diluted at 4 nM as a final concentration before being pooled. Finally, 15 pM of the denatured pool was paired end sequenced using the MiSeq Nano reagent kit v2 (2 × 250) (Illumina Inc).

Detailed information is reported in Text S1.

### 2.4. Consensus Sequences at Different Cutoffs and Viral Haplotypes

Fastq files were mapped against HCV consensus D90208 using BWA mem (available at https://github.com/lh3/bwa) [40] then BAM files were transformed into frequency files containing the numbers of observed nucleotides (A, T, C, G, N) along NS5A gene using bamToFreq [41].

Consensus sequences and the frequency of RAS were generated and analysed with prevalence cutoffs of 1%, 2%, 5%, 10% and 15% using geno2pheno [ngs-freq] [42] and Geno2pheno algorithm [39], respectively.

BAM files were also used to generate viral Haplotypes using QuasiRecomb [43].

### 2.5. Genetic Diversity Analysis

Nucleotide-sequence-variability (NSV) was estimated through an ambiguous nucleotide-positions percentage in NS5A ultra-deep consensus sequences using an in-house perl script. Genetic complexity was also evaluated as amino acid variability and this parameter was used to define NS5A heterogeneity. More specifically, the Shannon entropy weighted for intra-patient prevalence of viral haplotypes (Sn) was estimated for each NS5A amino acid position for each patient. The formula is reported in the Text S2.

### 2.6. Phylogenetic Analysis

#### 2.6.1. Genotype Assignment

For each patient, the HCV genotype and sub-genotype were determined. The NS3 + NS5A + NS5B sequences from Sanger sequencing were aligned and compared with reference sequences for all of the HCV genotypes and sub-genotypes (geno2pheno). The sequences were then manually edited using the Bioedit program, and gaps were removed from the final alignment. Genotype assignments were achieved by constructing phylogenetic trees, first using the Neighbor-Joining (NJ) method [44]. Distances were calculated using MEGA 6 based on the Kimura-2 parameter (K2P) model [45]. The robustness of the genotyping assignments was further tested using the Maximum Likelihood (ML) method. The ML tree was inferred using the General Time-Reversible nucleotide substitution model (GTR) with gamma-distribution among site rate heterogeneity, a proportion of invariable sites (GTR+I+Γ_5_) [46], and 1000 bootstrap replicates (Mega 6). The tree was rooted using a midpoint rooting with FigTree software v 1.4.4 (Rambaut A, Institute of Evolutionary Biology, University of Edinburgh, UK) [47].

#### 2.6.2. Identification of Transmission Clusters

Transmission clusters were first deduced using a Maximum-Likelihood tree as described above by using NS5A sequences from Sanger sequencing and NGS. The robustness of the transmission clusters was further tested using a Bayesian analysis. The Bayesian phylogenetic tree was reconstructed with MrBayes [48], using a GTR + I + Γ_5_. The Monte Carlo Markov Chain (MCMC) search was run for 5 × 10^6^ generations with the trees sampled every 100th generation (with a burn-in of 50%) [49]. Statistical support was obtained by calculating the posterior probability of each monophyletic clade, and a posterior consensus tree was generated after 50% burn-in. Clades were considered epidemiological clusters only if a posterior probability ≥90 was inferred.

#### 2.6.3. Statistical Analysis

Statistically significant differences were calculated using Fisher′s exact test and the Mann–Whitney test using R software (R Core Team, Vienna, Austria, available at: http://www.R-project.org/) [50].

## 3. Results

### 3.1. Patient Characteristics

This study included 28 HCV infected patients followed in two Italian hospitals (15 males, 53.5%) with a median age of 43 years [IQR 34–52]. For diagnostic purposes, a genotypic test based on the Sanger sequencing of NS3, NS5A and NS5B was performed. All patients were infected with GT1b. Median log serum HCV-RNA was 4.9 [IQR 3.3–6.0] IU/mL, while median [IQR] ALT and AST values (available only for the patients with acute infection) were 485 [IQR 255–718] IU/L and 271 [IQR 85–547] IU/L, respectively. The rate of IL-28B rs12979860 genotype was CC (50.0%), CT (39.2%) and TT (7.1%). The clinical and virological characteristics of the 28 patients included in the study are shown in Table 1. 

### 3.2. NS3-NS5A-NS5B Sanger Sequences and NS5A NGS Sequences

Among the 28 patients analysed by Sanger sequencing, NS5B, NS3 and NS5A sequences were obtained for 23, 22 and 21 patients, respectively. The NS5A ultra-deep-sequencing was then performed in 15 patients with a positive amplification product, nine with acute infection and six with chronic infection, all putatively involved in TCs harboring HCV NS5A resistance.

The NGS yielded 4,627,078 reads with a median of 186,381 [IQR 157,296–204,495] reads per sample. For each patient, five consensus ultra-deep sequences with a prevalence cutoff of 1%, 2%, 5%, 10% and 15% were obtained by geno2pheno, and NS5A haplotype sequences were constructed by Quasirecomb. NGS confirmed infection with GT1b in all individuals. No cases of other genotype/subtype coinfections were present, with no additional minority non-GT1b variants, at all NGS cutoffs used. 

### 3.3. Cluster Identification

**Figure 1:**
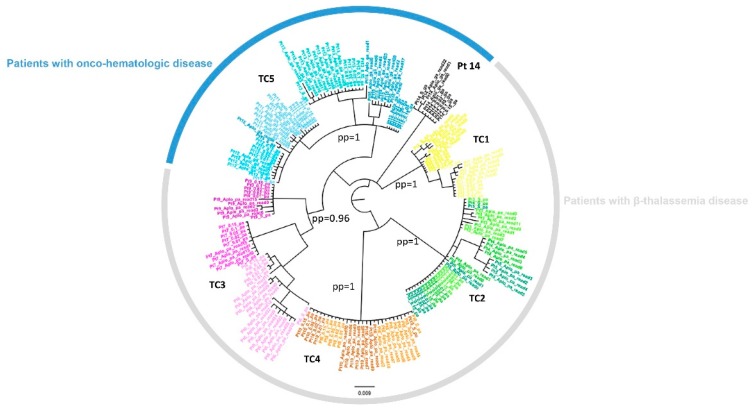
NS5A Bayesian Tree. The NS5A phylogenetic tree following the Bayesian method. The colors represent the different transmission clusters. Pt 14 was outside TC5. Transmission clusters (TC) were identified by a posterior probability ≥0.90.

To characterize the transmission clusters, a Bayesian analysis was performed in 15 patients by using both Sanger and NGS NS5A sequences. Specifically, for NGS NS5A sequences, haplotypes and five different consensus sequences (obtained at cutoff of 15%, 10%, 5%, 2% and 1%) were used for each patient. Among the 15 patients analyzed, five transmission clusters involving 14 patients were identified: four onco-hematologic patients with HCV acute infection, four β-thalassemia patients with acute infection and six β-thalassemia patients with chronic infection. In onco-hematologic patients, only one transmission cluster was observed involving four HCV acute patients (Pt11-Pt12-Pt13-Pt15) while one patient (Pt14) was not involved in the cluster. The other transmission clusters involved patients with β-thalassemia. One cluster involved three HCV chronic patients (Pt6-Pt7-Pt9) and three transmission clusters included both acute and chronic HCV infected patients (Pt1-Pt2; Pt3-Pt4-Pt5; Pt8-Pt10). Bayesian phylogenetic trees for the two groups of patients are shown in Figure 1.

### 3.4. NS3-NS5A-NS5B RASs in Sanger Sequences and in NS5A NGS Sequences

Sanger sequencing showed no RASs in any NS3 and NS5B genes analyzed, with the exception of S122T, a polymorphism associated with resistance to NS3-inhibitors, which was found in four onco-hematologic patients, forming cluster 5. Instead, in the NS5A gene, the R30Q, L31M and/or Y93H were detected by both sequencing analyses in seven patients (Table 2).

Using NGS, no additional minority NS5A-resistant variants were identified at any of the cutoffs used. The NS5A-RAS Y93H was observed in seven out of 15 patients analyzed. Particularly, three out of nine patients (33.3%) with acute infection and four out of six patients (66.6%) with chronic infection presented RAS. This mutation was observed in all five transmission clusters, even if it was not shared by all the patients involved in the same cluster. In transmission cluster 2, the β-thalassemia patient with chronic HCV infection showed the Y93H at 32.9% of intra-patient prevalence, but this mutation was not present in the other two HCV acute patients, involved in the same cluster (NGS cutoff up to 1%).

In contrast, two transmission clusters showed the same RAVs with a similar intra-patient prevalence among all individuals with both acute and chronic HCV infection. More specifically, cluster 1 showed the Y93H with a prevalence >97.8% in both Pt1 and Pt2, and cluster 4 showed a pattern of multi-resistance R30Q, L31M and Y93H with high prevalence of >97%, in both Pt8 and Pt10. Pt8 with chronic HCV infection was the only patient with a previous DAA-failure. 

Finally, in cluster 5, only one HCV acute onco-hematologic-patient showed the Y93H at 99.2% of intra-patient prevalence, but this mutation was not present in the other three HCV acute patients involved in the same transmission cluster (Table 2).

### 3.5. Common Patterns of Variants Transmitted within the Same Cluster

Furthermore, in order to better characterize the variants transmission, we analysed common patterns of variants shared between patients within the same cluster. For each cluster, common patterns of variants were identified (see Table S1). The number of mutations and common patterns differed according to the different cutoff of detection of minority variants used and whether the cluster was composed only of patients with acute HCV infection, acute and chronic HCV infection, or only chronic HCV infection. A higher number of mutations was observed in chronic HCV infected patients at 1% cutoff (a median of 20.5 mutations vs a median of 13 mutations in acute patients). A higher number of common patterns was observed in clusters made up of patients with only acute or acute and chronic HCV infection at a 15% cutoff (>81.82% vs. 56.21 in the cluster of only chronic HCV infected patients). Interestingly, cluster 4, containing one acute patient and the only chronic patient who had previously failed a DAA regimen, was the cluster with the highest percentage of sequence similarity within the common patterns (81.82% at 1% cutoff and 100% at 15% cutoff) whereas cluster 3, composed of only chronic patients, was the cluster with lowest percentage of sequence similarity (39.11% at 1% cutoff and 56.21% at 15%) (Table 3).

### 3.6. Nucleotide Sequence Variability and Shannon Entropy

**Figure 2:**
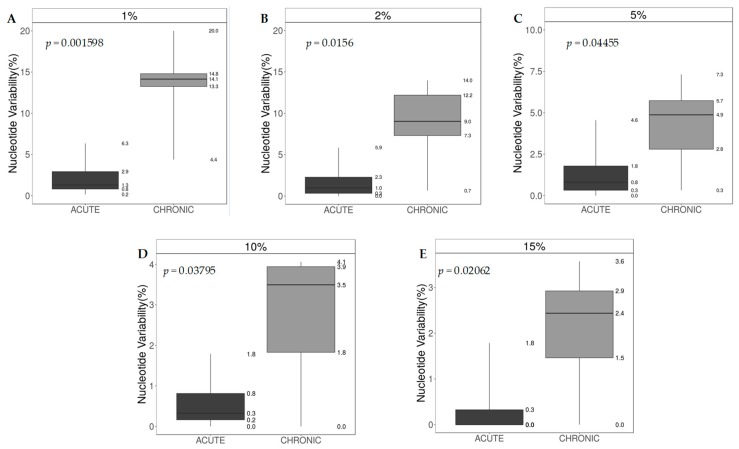
NS5A nucleotide sequence variability (%), Acute vs Chronic. Difference between acute and chronic infected patients in Nucleotide-sequence-variability at different NGS cutoff of detection of minority variants. The *p*-value was calculated using the Mann-Whitney U Test. (**A**) nucleotide sequence variability at 1%; (**B**) nucleotide sequence variability at 2%; (**C**) nucleotide sequence variability at 5%; (**D**) nucleotide sequence variability at 10%; Panel E: nucleotide sequence variability at 15%.

Generally, the intra-host HCV nucleotide sequence variability (NSV) obtained by using all NGS prevalence cutoffs (1%, 2%, 5%, 10%, 15%) was always higher in patients with HCV chronic infection vs patients with HCV acute infection (*p* < 0.05), with the highest difference at 1% cutoff (median [IQR] NSV 14.1 [13.3–14.8] in patients with chronic HCV infection vs 1.3 [0.8–2.9] in patients with acute HCV infection, *p* = 0.0016) (Figure 2).

The Shannon entropy showed a similar trend, with generally higher values in patients with chronic HCV infection vs patients with acute HCV infection, however, significantly higher values were observed only when comparing β-thalassemia patients with chronic infection versus β-thalassemia patients with acute HCV infection (Sn median HCV chronic 0.66 [IQR 0.60–0.81] vs Sn median HCV acute 0.34 [IQR 0.27–0.36] *p* = 0.01). In contrast, no significant difference was observed between Sn values from HCV acute onco-hematologic-patients vs HCV chronic β-thalassemia-patients (Sn median 0.55 [IQR 0.54–0.60] and Sn median 0.66 [IQR 0.60–0.81], respectively).

Interestingly, different Shannon Entropy median values were also observed between HCV sequences of acute onco-hematologic-patients vs HCV sequences of acute β-thalassemia-patients (Sn median 0.55 [IQR 0.54–0.60] vs median 0.34 [IQR 0.27–0.36] *p* = 0.02) (Table 4), indicating a higher HCV sequence heterogeneity in patients with HCV acute infection in the presence of an onco-hematology disease in comparison to patients with HCV acute infection in the presence of β-thalassemia. This observation was also confirmed as a significant trend by analysing the intra-host NSV between the two groups of HCV acute patients with the two different diseases (see Figure S1).

### 3.7. IL28B Polymorphisms and Intra-Host HCV Variability

To better characterize a potential virus-host interaction, we also investigated the relationship between human polymorphisms IL-28B rs12979860 and the presence of Y93H, the nucleotide sequence variability (NSV) and the Shannon entropy. Among the 15 patients analysed, the IL-28B information was available for 14 patients (13 of whom were involved in the clusters, see Table 2). Overall, CC polymorphism was present in seven patients and CT polymorphism in the remaining seven patients. No specific relationship between IL-28 (CC and CT) and Y93H, NSV and Shannon Entropy were identified with any statistically significant difference (*p* > 0.05; data not shown).

## 4. Discussion

The use of molecular methodologies based on viral gene sequencing and phylogenetic analysis is the best approach to identify and confirm potential suspected viral transmission events, by assessing genetic viral similarities within the individuals allegedly involved. A number of studies have shown that a phylogenetic tree analysis of individual HCV RNA isolates provides clear documentation of HCV transmissions occurring within healthcare settings. In 2006, molecular phylogenetic analysis was used to prove the innocence of foreign medical staff accused of transmitting HIV strains to children being treated in a hospital in Benghazi, in Libya [30]. In that study, in fact, researchers showed that both HCV and HIV-1 strains were already circulating in the hospital, before the arrival of the foreign medical staff in March 1998, suggesting that the hospital had a long-standing nosocomial infection control problem [30]. Several other nosocomial HCV transmission scenarios have been observed, with HCV infection transmission between haemodialysis patients [51,52,53,54,55,56], or among hematologic patients, generally through inappropriate use of medical devices (e.g., the inappropriate reuse of saline flush syringes) [57] and in patients with β-thalassemia [58,59]. However, the number of reported transmissions during the acute phase is limited, mainly because acute HCV infections are not always detected, due to the asymptomatic nature of the infection. Recently, an outbreak of HCV transmission in a Dutch haemodialysis unit was documented through the use of NS5A gene sequencing [10]. We have also recently identified separate episodes of HCV transmission in two healthcare settings by performing a phylogenetic analysis on NS3, NS5A and NS5B genes using Sanger sequencing: one case regarding patients with acute HCV infection diagnosis affected by onco-hematologic disease [36] and the second case in patients with acute HCV infection diagnosis with β-thalassemia [31]. In this present study, through NS5A NGS analysis we aimed to investigate the intra-host viral quasispecies composition in 15 patients, all of whom were putatively involved in transmission clusters. The Bayesian analysis on NS5A gene confirmed the five transmission clusters considered epidemiologically monophyletic clades, by a posterior probability ≥90. Among the 15 patients analyzed, 14 patients were involved in transmission clusters, composed of between two and four patients per cluster. Among the five onco-hematologic patients, all of whom showing acute HCV infection in the same hospital during the same period, the NGS analysis confirmed a transmission cluster involving only four patients, with one patient outside the cluster, for whom no evidence of minority HCV variants in common with the other four patients was found. The other transmission clusters concerned β-thalassemia patients with acute and/or chronic HCV state of infection. Notably, three transmission clusters involved both acute and chronic HCV infected patients and one cluster involved three HCV chronic patients, all of whom were being treated in the same clinical center, during the same period. NGS analysis confirmed that all individuals were infected with HCV GT1b subtype, without cases of minority variants of other genotypes and/or HCV subtypes, excluding cases of coinfection. Overall, the Bayesian phylogenetic tree performed on NGS NS5A sequences confirmed the previous results, suggesting that the viruses present in the identified clusters have such a high degree of similarity that they can be assumed to have a common origin.

Specific NS5A RASs (R30Q, L31M and/or Y93H) were previously observed using Sanger sequencing in all five transmission clusters. Through NGS, the patterns of resistance were confirmed, and no additional minority NS5A-resistant variants were identified at any of the cutoffs used (up to 1%). In all clusters at least one patient showed the Y93H, even if it was not shared within all patients involved in the same transmission cluster. This RAS was present overall in 37.5% of patients with acute HCV infection and in 66.7% of patients with chronic HCV infection. This prevalence is quite high, in all probability due mainly to these existing nosocomial transmission events. In fact, this level of prevalence is higher than the natural prevalence recently reported in a large Italian cohort of DAA naïve patients infected with HCV GT1b, which was around 10% [23]. In one cluster (TC2) the patient with chronic HCV infection showed the Y93H at 32.9% of intra-patient prevalence, and this mutation was not present in the other two patients with acute HCV infection, who were in the same transmission cluster. In this case, it can be presumed that the wild-type variant from the individual with the chronic infection (around 70% of intra-patient prevalence) was the major variant transmitted in the two individuals who acquired the acute HCV infection. In contrast, the patients with acute HCV infection found in transmission clusters TC1 and TC4 showed the same RAVs, with the same high intra-patient prevalence rate, as observed in the patients with chronic HCV infection belonging to the same clusters (>97.0%). In both cases, there is virological evidence of resistance transmission. For instance, in TC4, the patient with a chronic infection (Pt8) had previously failed a DAA treatment containing a NS5A inhibitor (grazoprevir/elbasvir) and developed at failure a resistant HCV with three NS5A RASs (R30Q, L31M and Y93H), which was fully transmitted to the patient who acquired the acute HCV infection (Pt10). Even if this case is, to our knowledge, the first case of nosocomial transmission of an HCV resistant variant, it is important to mention that a case of the transmission of an HCV NS3 resistant variant from a telaprevir treated patient to his sexual HIV positive partner has already been observed. [60].

To uncover the potential mechanisms of the intra-host variability observed within the nosocomial transmission clusters identified, we investigated the relationship between Y93H and the IL-28 polymorphisms in the rs12979860 gene. A previous study analysing the prevalence of resistant variants in a large European population of HCV infected individuals, in correlation to SNPs in IFNL4, in fact showed that the NS5A resistant variant Y93H was detected frequently in HCV GT 1b (14%) and it was significantly associated with the beneficial rs12979860 IL-28B polymorphism CC [61]. In our study, however, probably due to the limited numbers of cases analysed, even though all the individuals were infected with HCV GT 1b, we did not find any specific and statistically significant association between Y93H and the IL-28B polymorphism CC. 

To better characterize the virus host interactions, we also analysed the HCV variability and Shannon entropy in individuals with acute HCV infection in comparison to individuals with chronic infection. As expected, the NSV was significantly higher in patients with chronic HCV infection versus patients with acute infection, at all cutoffs tested (*p* < 0.05), with the highest difference at 1% cutoff, indicating that HCV infection over a longer period of time led to higher levels of viral evolution and quasispecies diversity.

Analyzing both groups (acute vs chronic infection), with respect also to the underlying disease (β-thalassemia vs onco-hematologic disease), the HCV sequence heterogeneity, measured as Shannon Entropy, was significantly higher in chronic HCV patients in comparison to patients with acute infection, but only in the presence of β-thalassemia (*p* = 0.01). Significantly higher Shannon entropy was also observed analysing the sequences of HCV acute patients with onco-hematologic disease respect to HCV acute patients with β-thalassemia. Instead, similar levels of sequence heterogeneity were observed between HCV acute patients with onco-hematologic disease and chronic HCV patients with β-thalassemia. 

This observation can be explained by the compromised immune system of patients with onco-hematologic disease, who did not in fact develop anti HCV antibodies after the infection (also at the last follow-up visits). These patients therefore fought the spread of the virus less efficiently, thus allowing it to change more easily with high replication turn-over, similar to that which would occur in a patient with a chronic infection. For instance, the HCV-RNA levels corresponding to the samples of HCV sequencing of the acute patients with onco-hematologic disease were higher than the levels observed in the acute HCV infected patients with β-thalassemia. On the other hand, the patients with β-thalassemia during the acute infection were under strong immune pressure, effectively limiting the viral replication over such a short period, and as a consequence also limiting variability and heterogeneity. 

It is worth mentioning that the only β-thalassemia patient with HCV chronic infection that previously failed a DAA-treatment (Pt8) had a low value of Shannon Entropy, at similar levels to that observed in the other acute patients with β-thalassemia (Sn in Pt8 0.38 versus Sn median in HCV acute β-thalassemia patients 0.34 [IQR 0.27–0.36]) (Table 3), and this could be the consequence of the strong pharmacological pressure that allowed the selection and transmission of the resistant variants to the patient with acute HCV infection belonging to the same transmission cluster (TC4). Cluster TC4 was in fact the cluster with the highest percentage of common patterns at all of the cutoffs analysed (from 81.8% at 1% to 100% at 15%). 

In conclusion, even though this study is limited to a small number of individuals, we highlighted the presence of nosocomial transmission clusters in patients with a diagnosis of acute HCV infection. Transmission of treatment resistant HCV variants in some cases occurred, and the transmission/maintenance of Y93H RAS in acute infected individuals was independent of the IL-28 polymorphism. We also observed a differential degree of HCV sequence variability and heterogeneity, which was higher in patients with chronic HCV infection, followed by immunocompromised patients during acute infection, and lower in immunocompetent patients during the acute HCV infection. Further studies are needed to better understand the mechanisms driving the intra host interactions and the transmission/maintenance of resistance. 

## Figures and Tables

**Table 1:** Patient characteristics.

Patients, N		28
Patients with β-thalassemia, N		22
Patients with onco-hematologic disease, N		6
Male, N (%)		15 (53.5)
Age, Median (IQR)		43 (34–52)
HCV genotype		1b (100)
Patients with available rs12979860 IL-28B genotype, N (%)		
	CC	14 (50.0)
	CT	11 (39.2)
	TT	2 (7.1)
	Unknown	1 (3.5)
Acute Infection, N (%)		17 (61)
Diagnosis of Acute Infection, Year		2016
Chronic Infection, N (%)		11 (39)
Naive *		27
HCV-RNA (logIU/mL), Median (IQR)		4.9 (3.3–6.0)
AST (IU/mL), Median (IQR) **		271 (85–547)
ALT (IU/mL), Median (IQR) **		485 (255–718)

* 1 patient failed a DAA-based regimen for HCV. ** Values were calculated according to the information available (only for patients with acute infection). Abbreviations: IQR, interquartile range; IU, international unit; AST, aspartate aminotransferase; ALT, alanine aminotransferase.

**Table 2:** List of mutations found in each gene using Geno2pheno for both Sanger and Next-Generation Sequencing (NGS) sequences.

Cluster HCV	Pt	HCV Infection	HCV-RNA (IU/mL)	IL 28	RAS					Other Mutations			
					NS3 Sanger	NS5B Sanger	NS5A Sanger	NS5A NGS	% NGS	NS3 Sanger	NS5A Sanger	NS5A NGS 1%	NS5B Sanger
TC1	Pt1	Acute	3270	CT	None	None	Y93H	Y93H	99.7%	V48I, V51A, A66G, T72I, P86Q, K87A, V132I, F147S, V170I, S174T (5–180aa)	K6R, S17T, K26R, L34V, L37F, K78R, R123QR, V164AE, V174T, Q176M (1–184aa)	K6R, S17T, K26R, L34V, L37F, K78R, V164E, V174T, Q176M (1–205 aa)	T181N, S210A, C213S (153–337aa)
	Pt2	Chronic	2820000	CC	None	None	Y93H	Y93HY	97.8%	V48I, V51A, A66G, T72I, P86Q, K87A, V132I, F147LS, V170I, S174T (1–180aa)	K6R, S17ST, K26R, L34V, L37F, G49EG, K78R, V164A, V174T, Q176M (1–211aa)	K6R, V8IV, S17ST, K26KR, L34V, L37FL, G49EG, I63IL, K78R, C98CS, N105NS, R108KR, A114AS, N137NS, V138IMV, A146AT, V164AE, V174T, Q176MT (1–205aa)	S19NS, M57L, K81R, Q90K, R98K, N110NS, V116I, N117KN, K124E, Q127L, T181N, S210A, C213RS, S231NS, T377S, C451H, A513S, R531K (1–540aa)
TC2	Pt3	Chronic	2340000	CT	None	None	Y93HY	Y93HY	32.9%	S7A, V48I, Y56F, A66G, P86Q, K87S, S122N, V132I, F147S, A150V (2–180aa)	S3T, K6R, S17T, L34V, K44R, Q54H, T56IT, T64A, H85R, T122V, M133MV, V138LV, R157QR, V164A, V174T (1–213aa)	S3T, K6R, S17T, L34V, K44R, D50DE, I52IDNV, Q54H, T56IT, T64A, H85R, A92AT, T122AV, V124GV, M133MV, V138LIV, R157QR, V164A, V174T (1–205aa)	M57L, V85IV, Q90K, Q127L, N206K, K209A, A252AV, T377S, A513S, T520I, K523MR (1–548aa)
	Pt4	Acute	2090	CT	None	None	None	None	None	V48I, Y56F, A66G, P86Q, K87S, S122N, V132I, F147S, V170I (15–180aa)	S3T, K6R, S17T, L34V, K44R, Q54H, T64A, H85R, T122V, V138L, R157Q, V164A, V174T (1–187aa)	S3T, K6R, S17T, L34V, K44R, Q54H, T64A, H85R, T122V, V124GV, V138L, R157Q, V164A, V174T (1–205aa)	N206K, K209A, A252V, T377S, I424V, M426T, A513S, T520I, K523R (151–538aa)
	Pt5	Acute	165	CC	None	None	None	None	None	V48I, Y56F, A66G, P86Q, K87S, S122N, V132I, F147S, V170I (15–180aa)	S3T, K6R, S17T, L34V, K44R, Q54H, T64A, H85R, T122V, V138L, R157Q, V164A, V174T, C190CG (1–196aa)	S3T, K6R, V15AV, S17T, P32PS, L34V, K44R, Q54H, A61AV, T64A, T83MT, H85R, T122MV, V124GV, G132AG, V138L, R157Q, V164A, V174T, L199I (1–205aa)	N206K, K209A, A252V, R254KR, E258EQ, T377S, A513S, T520I, K523R, S549G, V552A (151–562aa)
TC3	Pt6	Chronic	1800000	CT	None	None	None	None	None	S7A, C16CW, V48I, S61A, A66G, P86Q, K87AS, F147S (1–180aa)	K6R, S17T, L34V, L37F, T56I, K78R, T79A, V164A, V174T, L183V, S201ST, M202MR, T213AT (1–213 aa)	K6R, S17AT, L34V, L37FL, Y43FY, Q54HQ, T56IT, I63FL, K78R, T79A, T83MT, N105NS, R108KR, V164AT, V174T, L183V, A197AT (1–205aa)	A15S, M57L, Q90K, N117R, R120N, Q127L, T130N, F162Y, G198K, N206NS, C213S, R254K, T377S, V405I, Q464E, V499T, A513S, R531K, S549G (1–565a aa)
	Pt7	Chronic	577000	CC	None	None	Y93H	Y93H	99.65%	S7A, V48I, V51A, S61A, A66G, T72I, P86Q, K87A, S122G, F147S, V170I (1–181aa)	K6R, S17T, L34V, L37F, K44R, G49EG, Q54H, K78R, H85N, V138I, V164A, V174T, Q176L, L183V (1–194 aa)	K6R, S17T, L34V, F36FL, L37F, K44R, G49EG, Q54H, T56IT, T64AT, V75AV, K78R, H85NS, V124GV, F127FS, V138I, K139KR, V164A, V174T, Q176L, Q181HQ, L183V (1–205aa)	A15S, M57L, Q90K, V116I, N117R, R120N, Q127L, T130N, V147IV, F162Y, S189PS, G198KR, C213S, R254K, T377S, V405IV, A421V, I424V, T427P, Q464E, V499T, A513S, T520MT, Q544R, S549G, L564V, S565P (1–569 aa)
	Pt9	Chronic	94600	CC	None	None	None	None	None	S7A, L14F, V48I, V51A, S61A, A66G, P86Q, K87A, F147S, S174A (1–180aa)	K6R, S17T, L34V, L37F, Q54H, V75A, K78R, T83M, Y161H, V164A, V174T (1–185aa)	K6R, I12IL, S17T, K26KR, L34V, P35LP, L37F, Q54H, V75A, K78R, C80CR, T83M, H85HCRY, A92AS, T99AT, P102LP, R108KR, Y161H, V164A, V174T, A197AT (1–205aa)	A15S, M57L, Q90K, N110S, V116I, N117R, R120N, Q127L, F162Y, K270R, T312S, L314S, V315A, A333AV, S335N, T377S, V405I, K441Q, Q464E, V499T, A513S, K535R, S549G (1–568aa)
TC4	Pt8	Chronic	219000	CT	None	None	R30Q L31M Y93H	R30Q L31M Y93H	98.8% 98.8% 99.1%	V48I, A66G, P86Q, K87A, F147S, A150V, I153V (1–180aa)	K6R, S17T, L34V, L37F, Q54H, K78R, R123Q, V124I, M133V, V164A, E171Q, V174T, Q176L, T204TP (1–210aa)	K6R, S17T, L34V, L37F, Q54H, N69NT, K78R, T95MT, R108KR, R123Q, V124I, M133MV, K139KR, V164A, E171Q, V174T, Q176L, A197T, L199V (1–205aa)	A39S, M57L, R65Q, Q90K, K106KR, S113G, Q127L, E131N, I134L, F162Y, S231N, I262V, T377S, A513S, R531K (1–568aa)
	Pt10	Acute	163	CC	None	None	R30Q L31M Y93H.	R30Q L31M Y93H	99.4% 99.4% 97.6%	V48I, A66G, P86Q, K87A, F147S, A150V, I153V (16–180aa)	K6R, S17T, L34V, L37F, Q54H, K78R, R123Q, V124I, M133V, V164A, E171Q, V174T, Q176LQ (1–177aa)	K6R, S17T, L23LP, L34V, L37F, Q54H, K78R, R123Q, V124I, M133V, G155EG, V164A, E171Q, V174T, Q176L, A197T, L199V (1–205aa)	F162Y, S231N, I262V, T377S, T403AT, A513S, R531K (153–550aa)
TC5	Pt11	Acute	334000	CC	None	None	None	None	None	S7A, L14F, S61A, T72TI, D103ND, R118RW, S122T (181aa)	K6R, S17T, L34V, L37F, T83M, V138I, V164A, V174T, A197T (1–213aa)	K6R, S17T, L34V, L37F, V46IV, V75FV, T83M, H85HFLY, V138I, V164A, V174T, A197T (1–205aa)	T19S, L31IV, L36M, L47Q, N117R, R120N, T130N, T132S, F162Y, G198K, E202D, A207T, A210S, A218S, N231S, A300S, V321I E333A, K355Q, Q464E, V499T, R510K, S549G (1–570aa)
	Pt12	Acute	16100	CC	None	None	Y93HY	Y93H	99.2%	S7A, L14F, S61A, S122T (181aa)	K6R, S17T, L34V, L37F, T55AT, T83M, P89LP, H128HY, V138I, V164A, V174T, A197T (1–206aa)	K6R, S17AT, L34V, L37F, M53MV, T83M, V138I, V164A, V174T, A197T (1–205aa)	T19S, L36M, L47Q, N117R, R120N, T130N, T132S, G198K, E202D, A207T, A210S, A218S, N231S, A300T, V321I, E333A, K355Q, E437KE, Q464E, V499T, R510K, Q514R, S549G (1–580aa)
	Pt13	Acute	19690	CT	None	None	None	None	None	S7A, V48I, S61A, S122T (1–140aa)	K6R, S17T, L34V, L37F, T56IT, T83M, A92T, T135A, V138I, V164A, V174T (1–186aa)	K6R, S17T, L34V, L37F, T83M, A92T, T135A, V138I, V164A, V174T, A197AT, T200AT (1–205aa)	A16TA, T19S, L36M, L47Q, R98K, N117R, R120N, , T130N, E131EG, T132S, F162Y, G198K, E202D, A207T, A210S, A218S, N231S, C242S, A300S, V321I, E333A, K355Q, K441Q, Q464E, V499T, R510K, S549G (1–570aa)
	Pt15	Acute	1620000	NA	None	None	None	None	None	S7A, L13LF, L14F, S42SF, S61A, S122T, S93SF, S133SF (181aa)	K6R, S17T, L34V, L37F, T83M, V138I, V174T, A197T (1–213aa)	K6R, S17T, L34V, L37F, Q54HQ, T83M, A92AT, N105NS, V124GV, T135AT, V138I, R157LR, V164AV, V174T, P192PS, A197T, D205DE (1–205aa)	A16T, T19S, L36M, L47Q, R98K, N117R, R120N, T130N, T132S, F162Y, T181N, G189K, E202D, A207T, A210S, A218S, N231S, A300S, V321I, G328EG, E333A, K335Q, K441Q, Q464E, V499T, R510K, S549G, W574L, L588S (1–589aa)

Abbreviations: TC, transmission cluster; Pt, patient; RAS, resistance associated substitution; NGS, next-generation-sequencing. Genotype 1b sequences were used as a reference. NS5A ultra-deep consensus sequences were reported as 1% cutoff.

**Table 3:** Common patterns transmitted within the same clusters according to different cutoff of minority variants

Cluster HCV	Pt	HCV Infection	Cut-off 1%		Cut-off 5%		Cut-off 15%	
			N Mutations	Common Patterns %	N Mutations	Common Patterns %	N Mutations	Common Patterns %
TC1	Pt1	Chronic	20	50.00	13	69.23	11	81.82
	Pt2	Acute	10		10		10	
TC2	Pt3	Chronic	20	70.00	16	81.25	16	81.25
	Pt4	Acute	14		13		13	
	Pt5	Acute	20		14		14	
TC4	Pt8	Chronic	22	81.82	20	90.00	18	100
	Pt10	Acute	20		18		18	
TC3	Pt6	Chronic	17	39.11	13	55.87	11	56.21
	Pt7	Chronic	23		15		15	
	Pt9	Chronic	21		14		12	
TC5	Pt11	Acute	12	70.33	10	80.72	10	85.91
	Pt12	Acute	11		11		11	
	Pt13	Acute	12		11		11	
	Pt15	Acute	17		13		10	

Abbreviations: TC, transmission cluster; Pt, patient. Common patterns within the same clusters have been analyzed at different cutoff of detection of minority variants, between acute vs chronic (clusters 1, 2, 4); or between chronic infected patients (cluster 3), or between acute infected patients (cluster 5).

**Table 4:** Amino Acid Variability was estimated for each NS5A amino acid position for each patient.

Patient ID	HCV Infection	Number Cluster	Sn ^a^	Overall HCV Infection	Sn Median (IQR)	*p* Value ^b^
**Patients with β-thalassemia**						
1	Acute	1	0.23			
4	Acute	2	0.35			
5	Acute	2	0.36	Acute	0.34 (0.27–0.36)	
10	Acute	4	0.31			
2	Chronic	1	0.65			0.01 ^c^
3	Chronic	2	0.93			
6	Chronic	3	0.66			
7	Chronic	3	0.80	Chronic	0.66 (0.60–0.81)	
9	Chronic	3	0.59			
8	Chronic	4	0.38			
**Patients with onco-hematologic disease**						
11	Acute	5	0.59			
12	Acute	5	0.54			
13	Acute	5	0.41	Acute	0.55 (0.54–0.60)	0.02 ^d^
14	Acute	Out of cluster	0.68			
15	Acute	5	0.53			

^a^ Sn, Shannon Entropy weighted for the intra-patient prevalence of viral species, ^b^ *p* value was calculated using the Mann–Whitney test. ^c^ Acute vs chronic; ^d^ acute vs acute.

## Supplementary Materials

The following are available online at https://www.mdpi.com/2073-4409/8/7/666/s1, Figure S1, Table S1, Texts S1-S2 [62,63].
