# Supplementary material for: NS5A Gene Analysis by Next Generation Sequencing in HCV Nosocomial Transmission Clusters of HCV Genotype 1b Infected Patients
Source: Cells. 2019 Jul 2;8(7):666. doi: 10.3390/cells8070666 (PMC6678654; doi:10.3390/cells8070666)
Supplement: Supplementary file 1 [file cells-08-00666-s001.pdf]

Article

NS5A Gene Analysis by Next Generation Sequencing in HCV Nosocomial Transmission Clusters of HCV Genotype 1b Infected Patients

Maria Concetta Bellocchi [1,†], Marianna Aragri [1,†], Luca Carioti [1], Lavinia Fabeni [1], Rosaria Maria Pipitone [2], Giuseppina Brancaccio [3,4], Maria Chiara Sorbo [1], Silvia Barbaliscia [1], Velia Chiara Di Maio [1], Fabrizio Bronte [2], Stefania Grimaudo [2], Walter Mazzucco [2], Ferdinando Frigeri [5], Marco Cantone [3], Antonio Pinto [6], Carlo Federico Perno [7], Antonio Craxì [2], Giovanni Battista Gaeta [3], Vito Di Marco [2] and Francesca Ceccherini-Silberstein [1,*]

1 Department of Experimental Medicine, University of Rome Tor Vergata, 00133 Rome, Italy
2 Health Promotion Sciences, Maternal and Infant Care, Internal Medicine and Medical Specialties (PROMISE) Department, University of Palermo, 90127 Palermo, Italy
3 Infectious Diseases, Department of Mental and Physical Health and Preventive Medicine, Campania University "Luigi Vanvitelli", 80138 Naples, Italy
4 Department of Molecular Medicine, University of Padua, 35121 Padua, Italy
5 Hematology Division, "San Sebastiano" Hospital, 81100 Caserta, Italy
6 Hematology Department, National Cancer Institute "Fondazione Pascale", IRCCS, 80131 Naples, Italy
7 Department of Microbiology and Clinic Microbiology, University of Milan, 20162 Milan, Italy
* Correspondence: ceccherini@med.uniroma2.it; Tel.: 0039-0672-596-566
† These authors contributed equally to this work.

Received: 9 April 2019; Accepted: 25 June 2019; Published: 2 July 2019

Supplementary Text 1

Ultra-deep-sequencing and libraries preparation

To prepare indexed libraries of NS5A gene from each sample for sequencing on MiSeq platforms, 10 μL HCV-RNA genomic was reverse-transcribed and amplified using SuperScript III One-Step RT-PCR System (Invitrogen, Life Technologies, Carlsbad, CA, USA) for a final volume of 50 μL and with an eventual Nested-PCR as previously described [1].

NS5A amplicon produced were purified using AMPure XP magnetic beads (Beckman Coulter, Brea, CA, USA) and quantified with Qubit HS dsDNA Assay Kit (Life Technologies, Carlsbad, CA, USA). After, from each sample 1 ng of amplicon produced was involved in a tagmentation reaction by Nextera XT DNA Library Kit (Illumina Inc, San Diego, CA, USA). Unique combination of an i5 and an i7 index primer was then added to each tagmented DNA sample. The index primers were attached to each sample via PCR cycles according to manifacture. Thereafter, the library obtained for each patient was purified again to remove very short library fragments and quantified to calculate the molar concentration. All libraries were normalized at 4 nM as final concentration and then were pooled in 5 μL. Finally, 15pM of pool denatured was sequenced using MiSeq Nano reagent kit v2 (2×250) paired end on Illumina MiSeq, using 6% of PhiX V3 (15 pM) as an internal control library.

Supplementary Text 2

Shannon Entropy Estimation

For each patient, the Shannon Entropy weighted for the intra-patient prevalence of viral species (Sn) was estimated for each NS5A amino acid position.

Considering the relative frequency of each amino acid p_i in the viral population, the IC expressed in bit units, is obtained by:

$$IC = log_2(20) + \sum_i p_i log_2(\pi) = 4.32 + \sum_i p_i log_2(\pi)$$

where π was calculated as K/m, where m is the length of the haplotype, K is indicative of the observed viral population diversity:

$$K = \frac{2}{n(n-1)} \sum_{i<j} w_i w_j d_{i,j}$$

with w_i and w_j being the population frequency of the i-th and j-th haplotype, $d_{i,j}$ being the amino acid differences between haplotype I and J, and n the number of haplotypes.

Sn values close 0 indicate the presence of single haplotype while values close 1 represent the presence of >1 haplotype with the same intra-patient prevalence. This parameter was used to defined a NS5A heterogeneity.

A position, in which only a unique amino acid occurs, has an IC of 4.32 bits. IC values close to 0 indicate the ideal condition in which all the 20 amino acids were detected at equal frequency (5%) at a specific position, and thus the highest degree of genetic-variability at a given position.

The above-mentioned formula was based on Ramirez et al. [2].

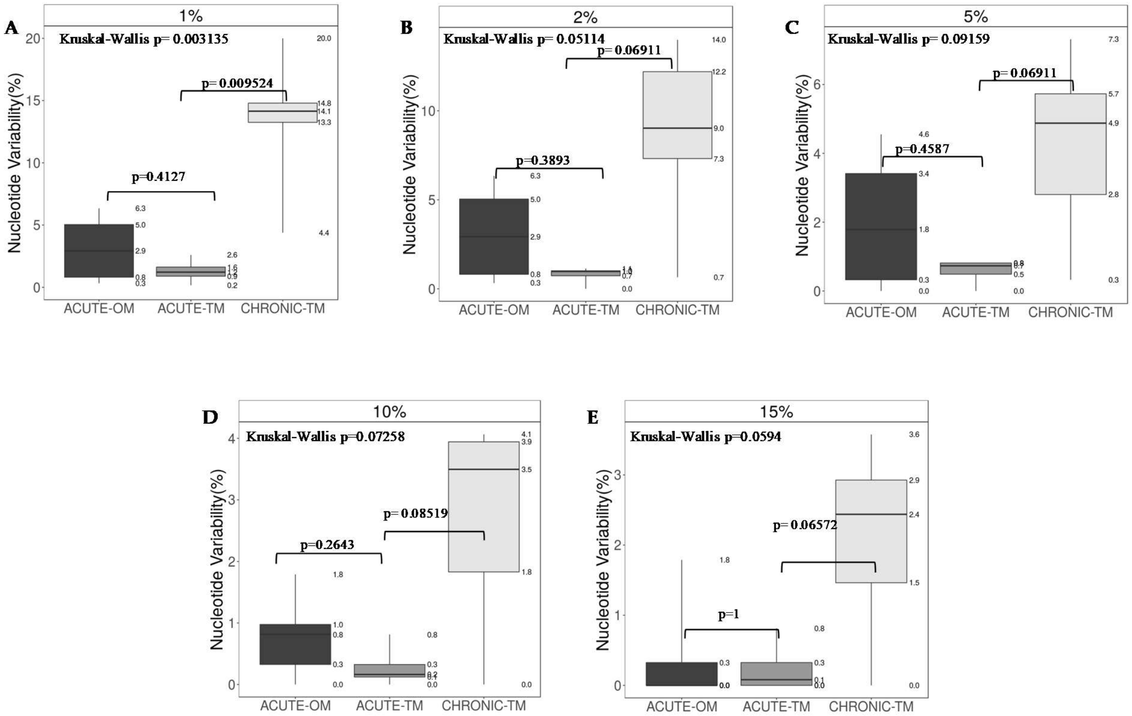

Supplementary Figure 1. NS5A nucleotide sequence variability (%) Acute vs Chronic. Difference between acute onco-hematologic (OM) infected patients, acute β-thalassemia (BT) infected patients and chronic β-thalassemia infected patients in Nucleotide-sequence-variability at different cut-off; p-value between AcuteOM vs AcuteTM and AcuteTM vs ChronicTM was calculated by Mann-Whitney U Test; p-value among AcuteOM, AcuteTM and ChronicTM was calculated by Kruskal-Wallis. Panel A: nucleotide sequence variability at 1%; Panel B: nucleotide sequence variability at 2%; Panel C: nucleotide sequence variability at 5%; Panel D: nucleotide sequence variability at 10%; Panel E: nucleotide sequence variability at 15%.

Supplementary Table 1. Common patterns at different next-generation-sequencing cutoff. .

**Cluster**	**Patterns (cutoff 1%)**
Cluster 1	K6R, S17T, K26R, L34V, L37F, K78R, **Y93H**, V164E, V174T, Q176M
Cluster 2	S3T, K6R, S17T, L34V, K44R, Q54H, T64A, H85R, T122V, V124GV, V138L, R157Q, V164A, V174T
Cluster 3	K6R, S17T, L34V, L37F, Q54H, K78R, V164A, V174T
Cluster 4	K6R, S17T, **R30Q, L31M**, L34V, L37F, Q54H, K78R, **Y93H**, R123Q, V124I, M133V, V164A, E171Q, V174T, Q176L, A197T, L199V
Cluster 5	K6R, S17T, L34V, L37F, T83M, V138I, V164A, V174T, A197T
	**Patterns (cutoff 5%)**
Cluster 1	K6R, S17T, K26R, L34V, L37F, K78R, **Y93H**, V174T, Q176M
Cluster 2	S3T, K6R, S17T, L34V, K44R, Q54H, T64A, H85R, T122V, V138L, R157Q, V164A, V174T
Cluster 3	K6R, S17T, L34V, L37F, Q54H, K78R, V164A, V174T
Cluster 4	K6R, S17T**, R30Q, L31M**, L34V, L37F, Q54H, K78R, **Y93H**, R123Q, V124I, M133V, V164A, E171Q, V174T, Q176L, A197T, L199V
Cluster 5	K6R, S17T, L34V, L37F, T83M, V138I, V164A, V174T, A197T
	**Patterns (cutoff 15%)**
Cluster 1	K6R, S17T, K26R, L34V, L37F, K78R, **Y93H,** V174T, Q176M
Cluster 2	S3T, K6R, S17T, L34V, K44R, Q54H, T64A, H85R, T122V, V138L, R157Q, V164A, V174T
Cluster 3	K6R, S17T, L34V, L37F,K78R, V164A, V174T
Cluster 4	K6R, S17T**, R30Q, L31M**, L34V, L37F, Q54H, K78R, **Y93H**, R123Q, V124I, M133V, V164A, E171Q, V174T, Q176L, A197T, L199V
Cluster 5	K6R, S17T, L34V, L37F, T83M, V138I, V164A, V174T, A197T

Resistance associated substitutions are highlighted in bold.

Reference

1. Di Maio VC, Cento V. Multiclass HCV resistance to direct-acting antiviral failure in real-life patients advocates for tailored second-line therapies. Liver Int 2017; 37: 514–28.
2. Ramírez C, Gregori J, Buti M, et al. A comparative study of ultra-deep pyrosequencing and cloning to quantitatively analyze the viral quasispecies using hepatitis B virus infection as a model. Antiviral Res. 2013;98:273-83.
